# Incidence of perioperative hypersensitivity reactions: A single-center, prospective, US cohort experience

**DOI:** 10.1016/j.jacig.2022.09.010

**Published:** 2022-10-30

**Authors:** Alexei Gonzalez-Estrada, Karol Avila-Castano, Joan M. Irizarry-Alvarado, Sher-Lu Pai, Gerald W. Volcheck

**Affiliations:** a Division of Pulmonary, Allergy and Sleep Medicine, Department of Medicine, Mayo Clinic, Jacksonville, Fla; b Division of General Internal Medicine, Department of Medicine, Mayo Clinic, Jacksonville, Fla; c Department of Anesthesiology and Perioperative Medicine, Mayo Clinic, Jacksonville, Fla; d Deparment of Allergic Diseases, Mayo Clinic, Rochester, Minn

**Keywords:** Incidence, hypersensitivity, anaphylaxis, POH, POA

## Abstract

**Background:**

A previous study, using administrative data, reported an incidence of perioperative anaphylaxis (POA) of 1:6537 procedures in the United States.

**Objective:**

We sought to determine the incidence of POA in a prospective US cohort.

**Methods:**

Adult participants undergoing a procedure at a single tertiary care center were studied prospectively between April 2018 and January 2022. Subinvestigators recorded vital signs and skin checks preoperatively, 15 minutes into induction, and hourly thereafter until 1 hour into the postoperative period. If participants developed an adverse reaction, additional variables were documented: causal agent(s) exposure, type of nonallergic adverse reaction, Sixth National Audit Project severity score, evidence of mast cell activation by serum acute and baseline tryptase pairing, Allergy consult, and causal agent identification.

**Results:**

Among 939 procedures (mean age, 59.25 ± 14.78 years; 58% females; 87% White), there were 12 (1.3%) cases with an identified adverse reaction. Nine cases were classified as nonhypersensitivity adverse reactions (1%) and 3 as possible hypersensitivity reactions (0.3%); 1 case was classified as suspected perioperative hypersensitivity and 2 as POA (0.2%). Both POA cases were males, had previous procedures, had evidence of mast cell activation, had a Sixth National Audit Project score of 3, and were referred to Allergy for further evaluation. There were 9 participants who developed a nonhypersensitivity adverse reaction: relative overdose of anesthetic (n = 6), transient rash (n = 2), and isolated bronchospasm (n = 1). All transient rashes were observed during undraping protocol.

**Conclusions:**

In our prospective study, the incidence of POA is 1:470 procedures. Our study suggests that the incidence of POA may be higher than previously reported.

## Introduction

The worldwide incidence of perioperative hypersensitivity (POH) ranges from 1:355 to 1:18,600 procedures, whereas the incidence of perioperative anaphylaxis (POA) ranges from 1:1,250 to 1:20,000 procedures.^1^ In the United States, the estimated incidence of POA is 1:6537 procedures based on administrative data.^2^ European survey-based, multicenter, cross-sectional, and prospective studies reported an incidence of POA between 1:736 and 1:2297 procedures.^3^^,^^4^ We sought to determine the incidence of POH and POA prospectively at our institution.

**Fig 1:**
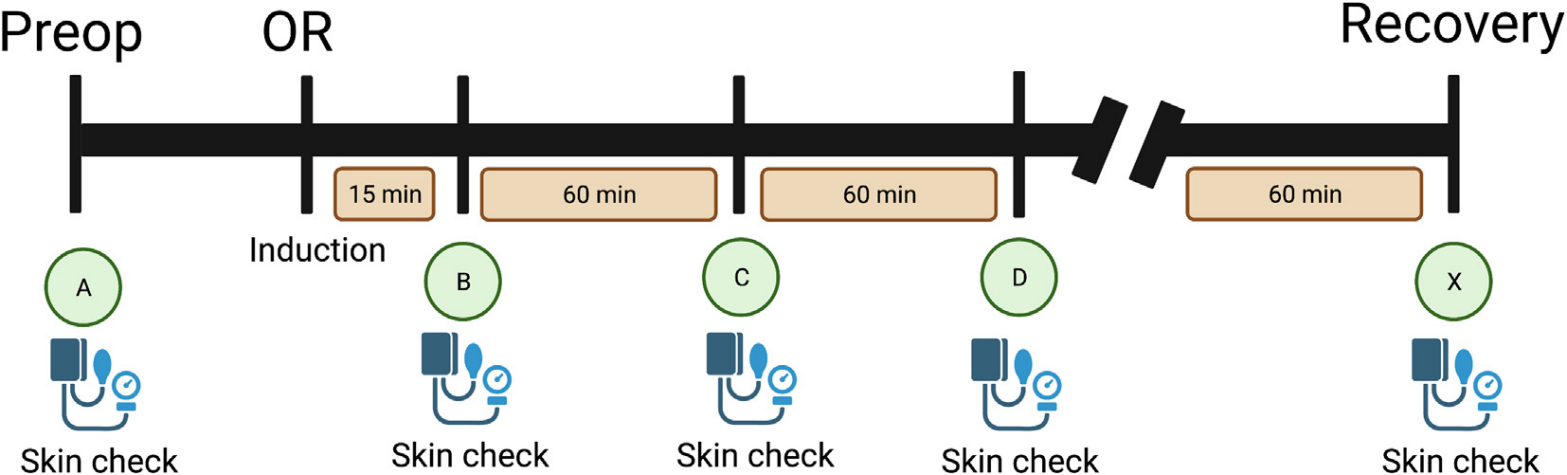
Graphical depiction of study protocol. Subinvestigators recorded vital signs and skin checks (undraping was performed if permitted by the OR team) preoperatively (*A*), 15 minutes into induction (*B*), and hourly thereafter (*C* and *D*, etc) until 1 hour into the postoperative period (*X*). *Preop*, Preoperatively.

With institutional review approval, adult participants undergoing a procedure (including surgery) provided verbal informed consent in the preoperative clinic or the preanesthesia holding area at our center between May 2018 and January 2022. Exclusion criteria included minors younger than 18 years and/or inability to consent. Demographic data and type of procedure were recorded. Subinvestigators provided observations of the participants in the operating room (OR). Vital signs and skin checks (undraping was performed when permitted by the OR team) were recorded on arrival at the OR (baseline), 15 minutes after the induction of anesthesia, and hourly thereafter until 1 hour into the postoperative period (Fig 1). If participants developed an adverse reaction, additional variables were documented: causal agent(s) exposure, adverse reaction characteristics, Sixth National Audit Project severity score,^5^ evidence of mast cell activation by serum acute and baseline tryptase pairing,^6^ management of index reaction, and Allergy consultation evaluation results. Adverse reactions were subcategorized into nonhypersensitivity reactions (see Table E1 in this article’s Online Repository at www.jaci-global.org)^7^ or POH, including POA defined per US guidelines.^8^ On the basis of a previous study,^4^ we categorized hypotension responsive to a vasopressor or isolated transient rash lasting for 3 minutes or less as nonhypersensitivity adverse reactions. Unexplained hypotension unresponsive to vasopressor or generalized urticaria was considered a hypersensitivity reaction. Descriptive statistics were performed via REDCap (Nashville, Tenn).

## Results and discussion

**Fig 2:**
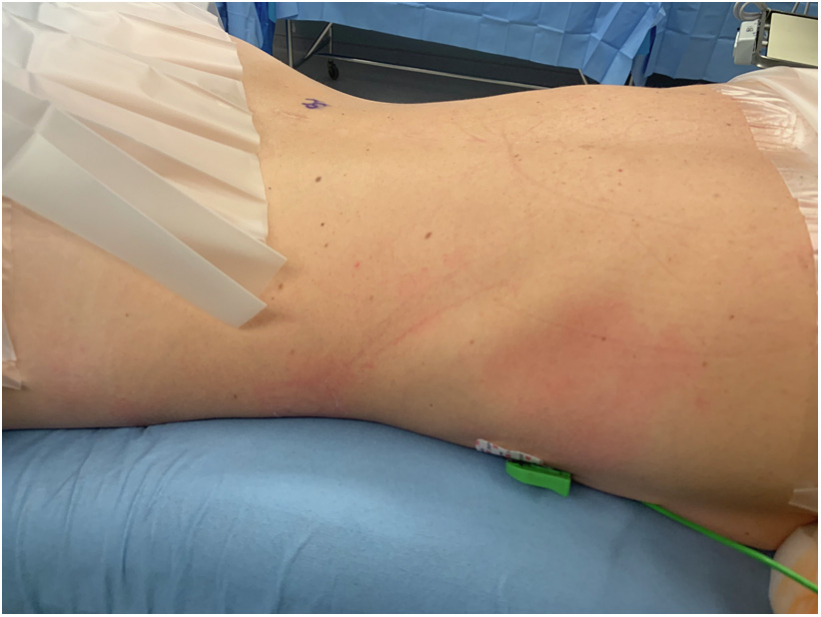
Photograph of participant’s upper and lower back revealing erythema, which lasted for approximately 10 seconds, thought to be secondary to fentanyl.

Of the 1906 eligible participants, 952 (50%) failed to complete protocol due to the following reasons: conflicting schedule with subinvestigator’s academic activities (59%), conflicting surgical schedule (28%), OR team declined investigator (12%), participant dropout (0.8%), or procedure cancelation (0.7%). Among the 939 participants included in this study (Table I), 12 (1.3%) developed an adverse reaction. Nine cases were classified as nonhypersensitivity adverse reactions (1%) and 3 as possible hypersensitivity reactions (0.3%). From the 3 cases with a possible hypersensitivity reaction, 1 case was classified as suspected POH and 2 as POA (0.2%). The nonhypersensitivity adverse reaction cohort (Table II) included 5 cases who developed transient hypotension, 2 who developed a transient rash (Fig 2), and 1 who developed isolated bronchospasm. Both POA cases were male patients who had previous procedures, had evidence of mast cell activation, had a Sixth National Audit Project score of 3, and were referred to allergy clinic for further evaluation. For the first POA case, the surgery was completed as scheduled, and the patient was later found to be allergic to cefazolin. The second POA case developed Kounis syndrome, had the surgery canceled, required intensive care unit stay, and was found to have latex allergy by serum specific IgE (Table III). In the single case of suspected POH (Table III), the participant developed face/neck erythema for 20 minutes and vital signs within normal limits, but the rest of the participant’s body remained draped throughout the procedure. Unfortunately, no referral to allergy was placed nor tryptase obtained for this single case.

**Table I:** Participant characteristics

Characteristic	Total (n = 939), n (%)
Age at procedure (y), median (IQR)	61.70 (50-70)
Female sex	545 (58)
Race	
White	818 (87.1)
Black	62 (6.6)
Asian/Pacific Islander	17 (1.8)
Other	17 (1.8)
Unknown/not specified	23 (2.4)
Ethnicity	
Not Hispanic or Latino	879 (93.6)
Hispanic or Latino	47 (5)
Not specified	13 (1.4)
Self-reported atopy	128 (13.6)
Allergic rhinitis	80 (62.5)
Asthma	49 (38.3)
Atopic dermatitis	9 (7)
BMI, median (IQR)	28.29 (25-32)
Obesity (BMI ≥30)	370 (39.4)
Previous surgery	886 (94.4)
No. of previous surgeries, median (IQR)	4 (2-6)
Type of anesthesia	
General	863 (91.9)
Monitored anesthesia care	39 (4.2)
Regional	24 (2.6)
Combined general and epidural	13 (1.4)
Participants undraped per protocol	759 (80.8)

*BMI*, Body mass index; *IQR*, interquartile range.

**Table II:** Characteristics of participants with nonhypersensitivity adverse reactions (n = 9)

Age (y)/sex	Atopy	BMI	Procedure performed/no. of previous procedures	Type of anesthesia/stage of protocol	Adverse reaction (lowest SBP or % change of baseline SBP)	Duration of reaction (min)	Suspected culprit agent	Management
67/F	No	35.0	Transplant/3	GA/1 h	Hypotension (32%) Undraping not allowed	10:02	Fentanyl Basiliximab	Ephedrine Phenylephrine
68/M	No	29.9	Skin/9	GA/15 min	Hypotension (79 mm Hg)	08:00	Propofol	Ephedrine Phenylephrine
69/M	No	21.8	CVS/4	GA/15 min	Hypotension (82 mm Hg)	59:00	Propofol	Permissive hypotension to decrease blood loss
37/M	No	27.8	Transplant/2	GA/1 h	Hypotension (80 mm Hg) Undraping not allowed	05:00	Propofol	Phenylephrine
77/M	Yes	29.0	ENT/6	GA/15 min	Hypotension (80 mm Hg)	22:00	Propofol	Ephedrine Phenylephrine
70/M	No	37.8	GI/3	Combined	Hypotension (76 mm Hg)	15:00	Propofol	Ephedrine
35/F	No	18.7	Neurological/6	GA/15 min	Upper and lower back erythema	00:10	Fentanyl	None
30/F	No	21.0	GI/6	GA/15 min	Face/neck erythema	02:00	Rocuronium Fentanyl	None
74/F	No	47.1	Hematologic/2	GA/1 h	Bronchospasm Undraping not allowed	05:00	Unknown	Albuterol

*BMI*, Body mass index; *GA*, general anesthesia; *GI*, gastrointestinal; *Combined*, GA and regional; *CVS*, cardiovascular; *ENT*, ears/nose/throat; *SBP*, systolic blood pressure.

**Table III:** Characteristics of participants with perioperative hypersensitivity (n = 3)

Characteristic	Perioperative anaphylaxis (n = 2)		Suspected perioperative hypersensitivity (n = 1)
Age (y)	55	52	74
Sex	Male	Male	Male
Race	White	Black	White
Ethnicity	Not Hispanic	Not Hispanic	Not Hispanic
Self-reported atopy	No	No	No
BMI	27.1	24.1	29.7
No. of previous surgeries	4	1	10
Type of anesthesia	MAC	GA	GA
Index reaction			
Signs	Hypotension Urticaria/angioedema Tachycardia	Hypotension Tachycardia Left ventricular dysfunction	Face/neck erythema lasting for 20 min. Unable to undrape rest of participant. The rest of the participant’s vital signs remained within normal limits
Anesthesia period	Maintenance	Maintenance	Induction
Severity score (NAP6)	3	3	1
Treatment	Epinephrine H1B CS IVFs	Epinephrine IVFs	None
Case outcome	Completed	Canceled, ICU care	Completed
Laboratory evaluation			
sAT (ng/mL)	14.9	223	ND
sBT (ng/mL)	6.3	20.3∗	ND
MCA	Present	Present	NA
Latex sIgE	Negative	Positive	ND
Skin testing results	Cefazolin + (IDT 3.3 mg/mL)	Negative	Rocuronium suspected. Not confirmed
Future procedure	No subsequent procedure	Pending (transplant)	NA

*BMI*, Body mass index; *CS*, corticosteroid; *GA*, general anesthesia; *H1B*, histamine receptor 1 blocker; *ICU*, intensive care unit; *IVF*, intravenous fluid; *MAC*, monitored anesthesia care; *MCA*, mast cell activation; *NA*, not applicable; *NAP6*, Sixth National Audit Project; *ND*, not done; *sAT*, serum acute tryptase; *sBT*, serum baseline tryptase.

∗ Elevated sBT secondary to end-stage renal disease.

We report the first prospective cohort study in the United States that followed 939 participants undergoing a procedure at a single tertiary care center. Among the 939 procedures, there were 12 (1.3%) cases with an adverse reaction, 3 (0.3%) of 939 or 1:313 with POH including 2 (0.2%), and 1:470, with POA. Previous prospective cohorts reported similar POH incidences to our study. Berroa et al^4^ reported 16,946 procedures, with anesthesiologists documenting all perioperative hypersensitivity reactions between February 2008 and August 2010. Cases that included isolated hypotension with an explanation and a transient generalized rash that lasted for 3 minutes or less were excluded. The study found an incidence of POH of 1:385 procedures and POA of 1:736 procedures.^4^ A cross-sectional survey-based study from the United Kingdom analyzed 4595 procedures from 12 hospitals during a 2-week period. The authors used the Association of Anesthetists of Great Britain and Ireland criteria for referral to a specialist, including unexplained hypotension, unexpected bronchospasm resistant to treatment, angioedema, urticaria, severe itch, and/or widespread erythema.^9^ The study found an incidence of 1:353 for POH and 1:2297 for POA. The authors concluded that POA may be underrecognized and underreported.^3^

The higher incidence of POA (1:470) in our study may be explained by the proactive undraping and vital sign documentation performed by subinvestigators. A possible explanation for the higher incidence of POA is the low denominator compared with the 2 previous studies. There is also a difference regarding defining POH versus an adverse reaction. Although most reports agree that an isolated transient rash should not be considered as POH, the allowed time for this transient rash reaction has not been specified in guidelines. We chose a time of 3 minutes or less on the basis of a previous report^4^; however, we acknowledge that this 3-minute cutoff is arbitrary and further international consensus is needed for a stricter transient rash time criterion.

There are limitations to our study. First, our findings were from procedures performed at a tertiary care center, and the results may not be generalizable to a community practice. A large proportion of participants did not complete the protocol mainly because of the time constraints on the completion of the protocol due to a limited number of subinvestigators. However, there was nothing to suggest that those participants who did not complete the protocol would have led to a difference in the clinical findings in the study. Finally, because not every participant underwent undraping during the procedure, the actual incidence of POH may be underreported.

In conclusion, we conducted a prospective study of 939 participants undergoing a procedure during a 4-year period. In this cohort, the incidence of POH was 1:313 and that of POA was 1:470 procedures. We speculate that POA remains underreported due to a lack of recognition in the OR because cutaneous hypersensitivity manifestations are hidden by surgical drapes and has a higher threshold for allergy referrals by anesthesia providers of suspected POA cases. Future multicenter prospective studies are warranted to establish the epidemiology of POH more accurately.

**Clinical implications**

In this prospective single-center cohort, the incidence of perioperative hypersensitivity was 1:313 procedures and of perioperative anaphylaxis was 1:470 procedures, suggesting that these conditions are likely underrecognized.
