# Supplementary material for: Incidence of perioperative hypersensitivity reactions: A single-center, prospective, US cohort experience
Source: J Allergy Clin Immunol Glob. 2022 Oct 30;2(1):88–92. doi: 10.1016/j.jacig.2022.09.010 (PMC10509856; doi:10.1016/j.jacig.2022.09.010)
Supplement: Supplementary Table 1 [file mmc1.docx]

| Table E1. Non-hypersensitivity mimickers of perioperative allergic reactions (normal serum tryptase) | |
| --- | --- |
| **Isolated angioedema or laryngeal/pharyngeal edema** | |
|  | Edema due to handling of difficult airway |
|  | Contact allergy to perioperative exposures (delayed onset 8-12h postoperative) |
|  | ACE inhibitor-elicited angioedema (onset 1-8 h after surgery) |
|  | Subcutaneous emphysema |
|  | Hereditary angioedema |
| **Isolated bronchospasm** | |
|  | Undiagnosed, untreated, or insufficiently treated asthma |
|  | Irritation from misplaced endotracheal tube, superficial/light anesthesia |
|  | Hyperreactive airways due to, e.g., Viral infections, smoking |
| **Isolated hypotension** | |
|  | Major bleeding |
|  | Relative overdose of anesthetic agents |
|  | Vasodilatory effect of neuroaxial blockade (spinal/epidural) |
|  | Treatment with tricyclic antidepressants |
|  | Amniotic fluid embolism/pulmonary embolism |
|  | Bone cement implantation syndrome |
|  | Other types of shock |
| **Isolated skin symptoms** | |
|  | Urticaria or angioedema in patients with existing chronic urticaria or angioedema |
|  | Nonspecific histamine release causing transient rash, flushing, itching |
| **Combination of tachycardia, flushing, hypotension** | |
|  | Nonspecific histamine release |
|  | Relative overdose of oxytocin |
|  | Mesenteric traction syndrome |
| With permission from Garvey LH. Chapter 21: Perioperative anaphylaxis. In: Khan DA, Banerji A, eds. *Drug allergy testing*. 1^st^ ed. Elsevier; 2018: 223-238 | |
